# Supplementary material for: Competing electronic states emerging on polar surfaces
Source: Nat Commun. 2022 Jul 25;13:4311. doi: 10.1038/s41467-022-31953-6 (PMC9314351; doi:10.1038/s41467-022-31953-6)
Supplement: Supplementary file 1 — Supplementary Information [file 41467_2022_31953_MOESM1_ESM.pdf]

Supplementary Information

Competing electronic states emerging on polar surfaces

Michele Reticcioli,[1,2] Zhichang Wang,[2,3] Michael Schmid,[2] Dominik Wrana,[4]
Lynn A. Boatner,[5] Ulrike Diebold,[2] Martin Setvin,[2,4] and Cesare Franchini[1,6]

[1]*University of Vienna, Faculty of Physics, Center for Computational Materials Science, Vienna, Austria*
[2]*Institute of Applied Physics, Technische Universität Wien, Vienna, Austria*
[3]*State Key Laboratory for Physical Chemistry of Solid Surfaces, Collaborative Innovation Center of Chemistry for Energy Materials, and Department of Chemistry, College of Chemistry and Chemical Engineering, Xiamen University, Xiamen, China*
[4]*Department of Surface and Plasma Science, Faculty of Mathematics and Physics, Charles University, 180 00 Prague 8, Czech Republic*
[5]*Materials Science and Technology Division, Oak Ridge National Laboratory, Oak Ridge, USA*
[6]*Dipartimento di Fisica e Astronomia, Università di Bologna, 40127 Bologna, Italy*

(Dated: June 16, 2022)

Supplementary Notes

Supplementary Note 1 Additional experimental data 2

Supplementary Note 2 Spin Degenerate 2DEG 9

Supplementary Note 3 Details of the Ferroelectriclike Distortions 10

Supplementary Note 4 Polaronic Configurations and Stability 11

Supplementary Note 5 The U parameter 13

Supplementary Note 6 CDW wavelength 14

Supplementary Note 7 Modeling the Phases of the Excess Charge 15

Supplementary References 15

Supplementary Figures

Supplementary Figure 1 States near the Fermi level . . . 3
Supplementary Figure 2 Detailed FFT of the wave pattern . . . 4
Supplementary Figure 3 STS of deeper states . . . 4
Supplementary Figure 4 Phase inversion of the CDW . . . 5
Supplementary Figure 5 Density of states above the Fermi level . . . 6
Supplementary Figure 6 Stability of the CDW . . . 7
Supplementary Figure 7 Spatial correlation between polarons and defects . . . 8
Supplementary Figure 8 Spin-degenerate 2DEG . . . 9
Supplementary Figure 9 Ferroelectriclike distortions . . . 10
Supplementary Figure 10 Sub-surface polarons . . . 11
Supplementary Figure 11 Bipolaron ordering . . . 12
Supplementary Figure 12 The U parameter . . . 12
Supplementary Figure 13 CDW coexisting with polarons . . . 14

Supplementary Note 1. ADDITIONAL EXPERIMENTAL DATA

Here we bring additional evidence for the presence of the charge density wave on the cleaved $KTaO_3$ surfaces. Supplementary Figure 1 shows maps of dI/dV measured at sample biases ranging from +0.1 to −0.2 V. The data was measured in two different regions of the same sample. The crystal was doped with 0.17% Ca for ensuring sufficient bulk conductivity. The images closest to the Fermi level show a clear wave pattern, which is reflected in the fast Fourier transform (FFT, right column). The dashed line in the panel for −0.025 V is a Fermi surface that could give rise to the CDW in a simple model with Fermi surface nesting. Detailed analysis of the wave pattern at energies further from the Fermi level shows a lack of dispersion, see Supplementary Figure 1. A detail of one of the Fourier transforms is shown in Supplementary Figure 2, together with a line profile along the (1,1) direction.

The corresponding wavelength of the magnitude of the wave function measured by STM is 1.55±0.15 nm, which is compatible with the Fermi level wave-vector of 2DEGs reported by ARPES [1, 2]: ARPES shows the Fermi k-vectors of 0.14 to 0.18 nm^{-1}, which would translate to STM peak-to-peak distances of the waves ranging from 2.2 to 1.7 nm.

The data both below and above the Fermi level keep the same periodicity of the wave, within the error bars. This means that the STS maps show only the CDW, not the dispersion of the 2DEG at lower energies as observed by ARPES [1, 2]. The same periodicity of waves is sometimes weakly observed at energies very deep in the band gap, which is illustrated in Supplementary Figure 3 at $V_S = -1.0$ and -1.64 V.

A key observation that indicates the presence of CDW is the phase inversion of images measured below and above the Fermi level [3]. This is shown in Supplementary Figure 4. The amplitude is inverted in most places when comparing the states just below and above the Fermi level. Supplementary Figure 4d,e mark positions where the inversion occurs by yellow dots and lines. Red dots and lines mark objects that do not develop the phase inversion.

The phase inversion is typically observed in larger TaO_2 terraces. It does not appear in the direction perpendicular to narrow terraces where the wave is confined to two or three oscillations. These regions develop the phase inversion only in direction along the steps, where the amplitude of oscillations is lower. It is likely that the features marked by red lines are standing waves originating from reflections of a 2DEG from the steps rather than the CDW. This would be consistent with the direction of these waves sometimes deviating from the standard $\langle 110 \rangle$ directions of the CDW.

As expected, these images show also that the CDW forms checkerboard patterns. This is related to the fourfold rotational symmetry with the [110] and [-110] being equivalent; however, the symmetry is broken by nearby KO-TaO_2 step edges, thus leading to different interference patterns.

For completeness, Supplementary Figure 5 shows maps of dI/dV measured above the Fermi level. Ony the states directly above the Fermi level show LDOS oscillations corresponding to the CDW.

An important characteristic of a CDW is that the stability is limited to a certain temperature range. This is shown in Supplementary Figure 6. The states directly below and above the Fermi level are shown, since they are characteristic for the CDW. The intensity of the states near the Fermi level decreases with the increasing temperature, and these states fade away above ≈ 80K. At $T = 51$ K, we can still recognize the wave pattern, but it is unclear whether it originates from standing waves or the CDW. We do not have a solid evidence about the phase inversion at elevated temperatures. The tunneling current originating from the in-gap states decreases by several orders of magnitude when comparing data measured at $T = 5$ K to data above 80 K. This observation was made systematically over a large number of experimental sessions.

The scenario of disappearance of the states close to the Fermi level is unconventional: Instead of a transition to a homogeneously distributed LDOS, the electronic states seem to disappear completely. This is apparent when comparing the magnitude of the tunneling current in experiments at different temperatures. We note that all experiments were performed with a copper-functionalized tip and the images were scanned at comparable height (estimated from the AFM images). The tunneling currents become dramatically lower at elevated temperatures and also the bottom of the conduction band shifts to slightly higher energies. This indicates that the electrons arising from the polar catastrophe may become unavailable at elevated temperature, which is likely linked to the destabilization of the CDW.

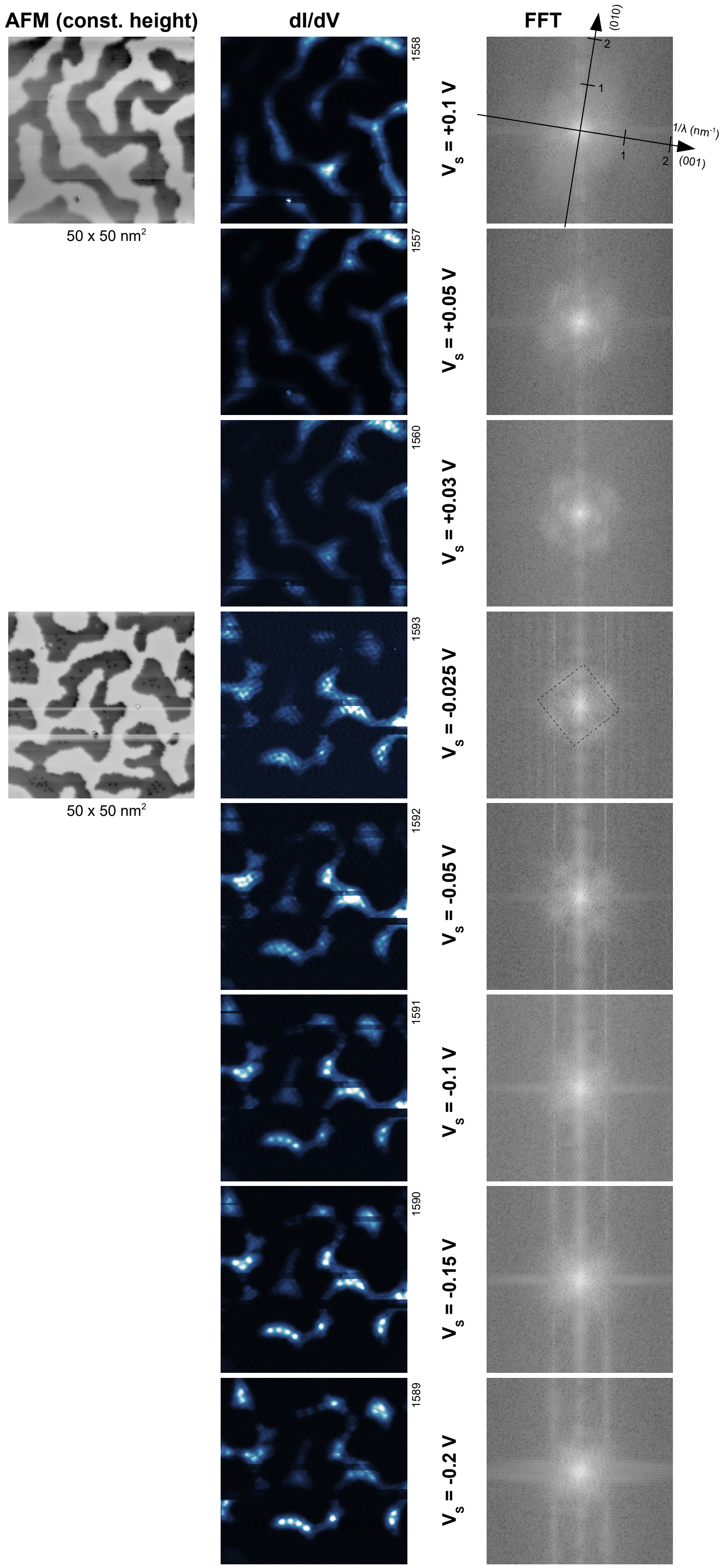

Supplementary Figure 1: **States near the Fermi level.** Constant-height dI/dV maps measured at various voltages near the Fermi level. The AFM images in the left column show the topographies where the data were acquired (two different regions on the same sample). The right column show the Fourier transform of the respective regions. The dashed line in the frame corresponding to $V_S = -0.025$ V shows a possible Fermi surface that could lead to the CDW by simple Fermi surface nesting.

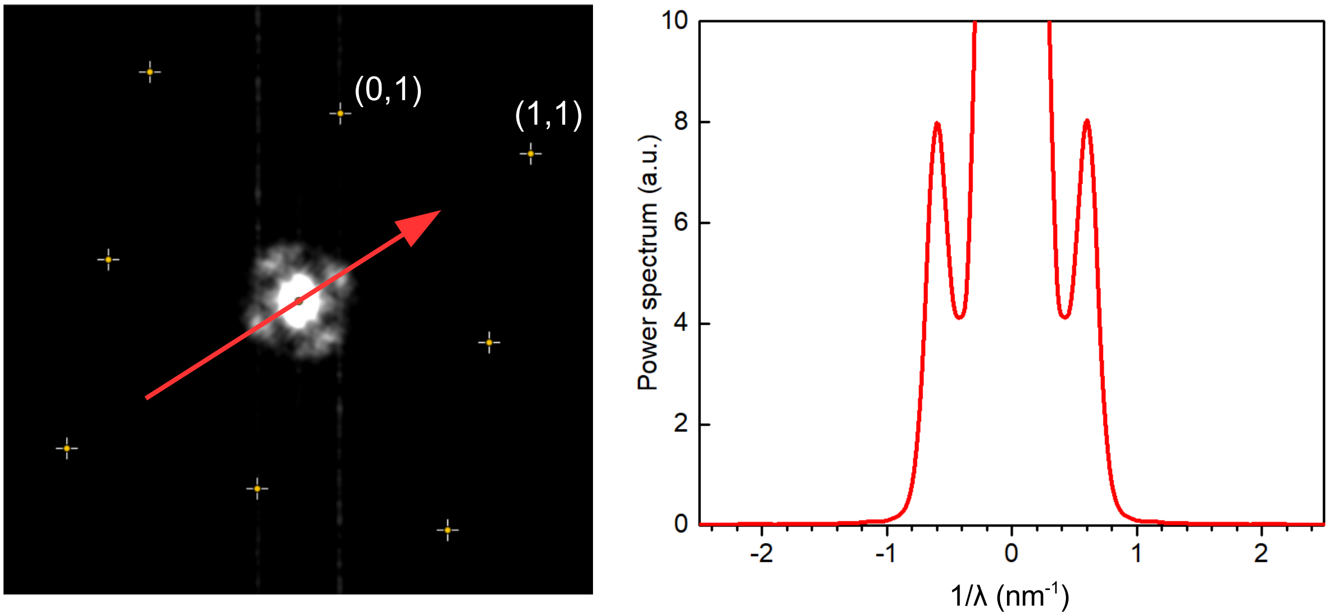

Supplementary Figure 2: **Detailed FFT of the wave pattern.** The left panel shows an FFT of the dI/dV map from Fig. SF1 corresponding to a sample bias of -25 mV. The spots of the reciprocal lattice are marked by crosses. A line profile along the red arrow is plotted in the right panel (the profile is averaged over 15 adjecent lines).

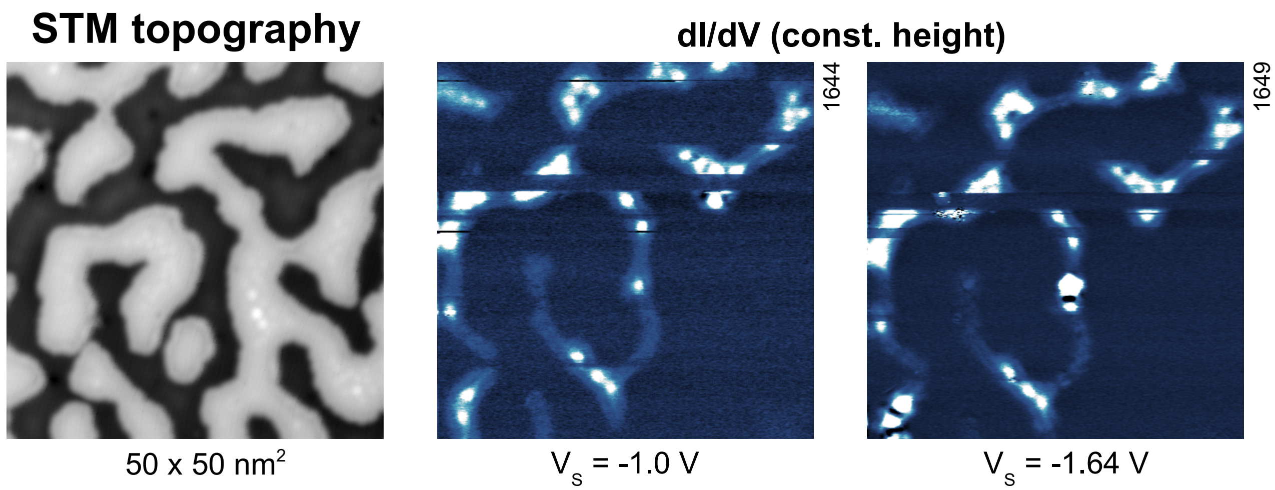

Supplementary Figure 3: **STS of deeper states.** Constant-height dI/dV maps measured deeper in the band gap. The left image shows an STM topography of the region where the data was acquired.

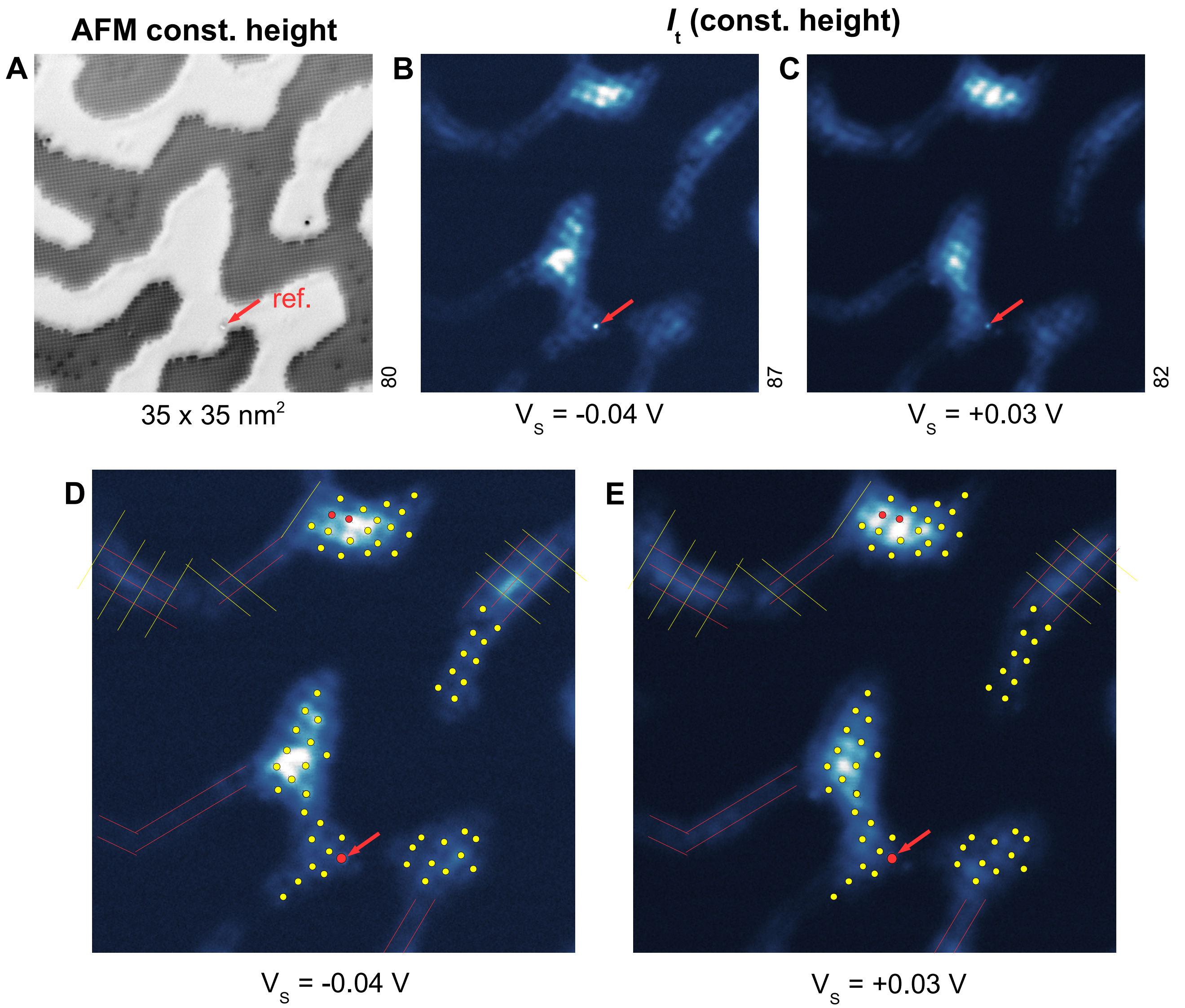

Supplementary Figure 4: **Phase inversion of the CDW.** a) Constant-height AFM image showing the layout of the KO and TaO_2 terraces. The red arrow marks a reference defect that is used for aligning all images. b,c) Constant-height tunneling current measured just below and above the Fermi level, respectively. d,e) The same images as b,c with marked positions. Dots and lines in d) mark maxima in the occupied states. The same positions are shown in the empty-states image in e). Yellow is used where phase inversion occurs, red marks the absence of phase inversion. We attribute the wave-like pattern in narrow terraces not showing phase inversion (red lines) to standing waves (quantum well states).

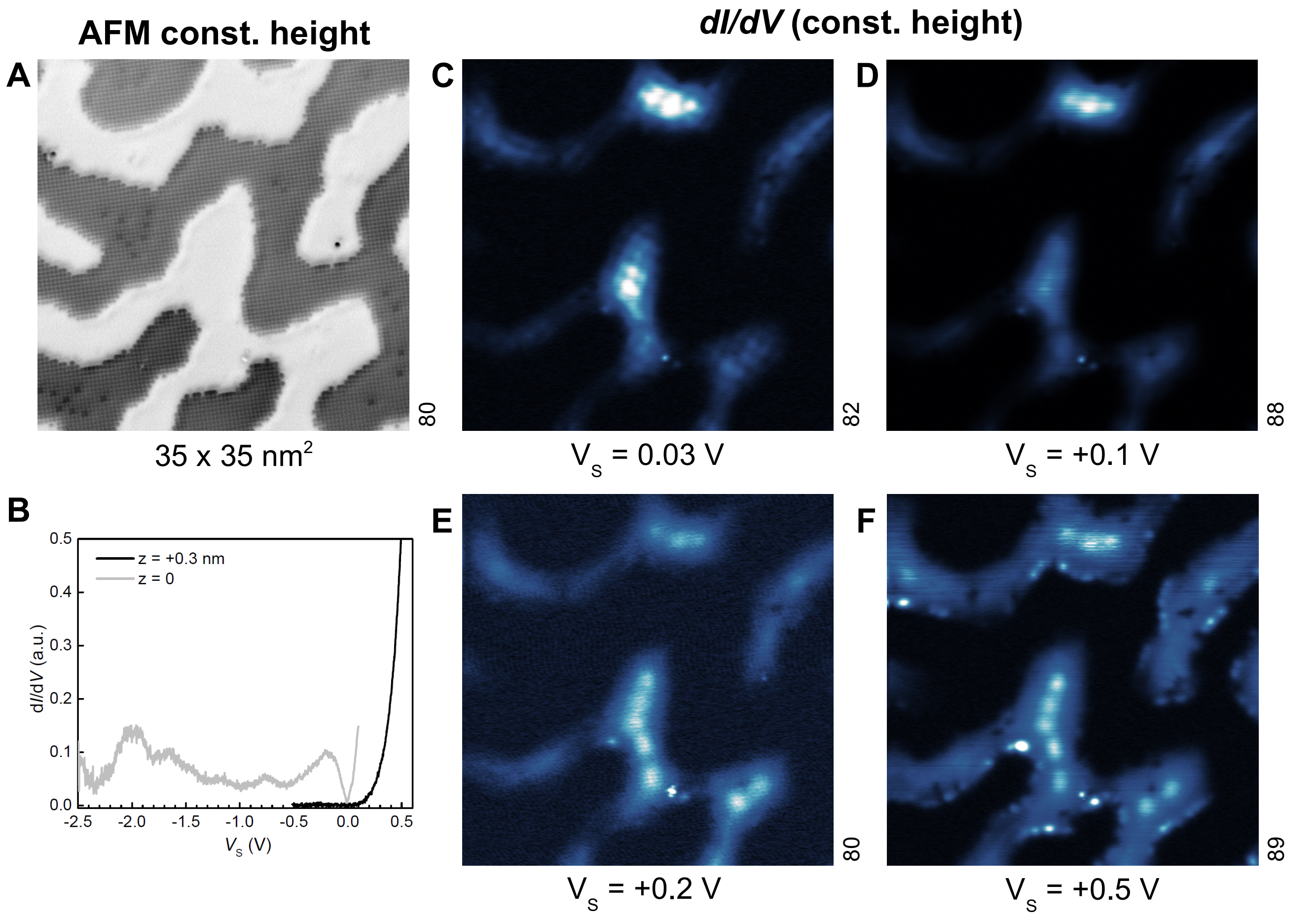

Supplementary Figure 5: **Density of states above the Fermi level** a) Constant-height AFM image showing the layout of the KO and TaO_2 terraces. b) representative dI/dV spectra measured in a central region of the TaO_2 termination. Grey line shows a spectrum measured at a close tip-sample distance, black line corresponds to data measured 0.3 nm further away, showing that empty states are dominated by the onset of the conduction band. c-f) Maps of dI/dV measured at different voltages in empty states.

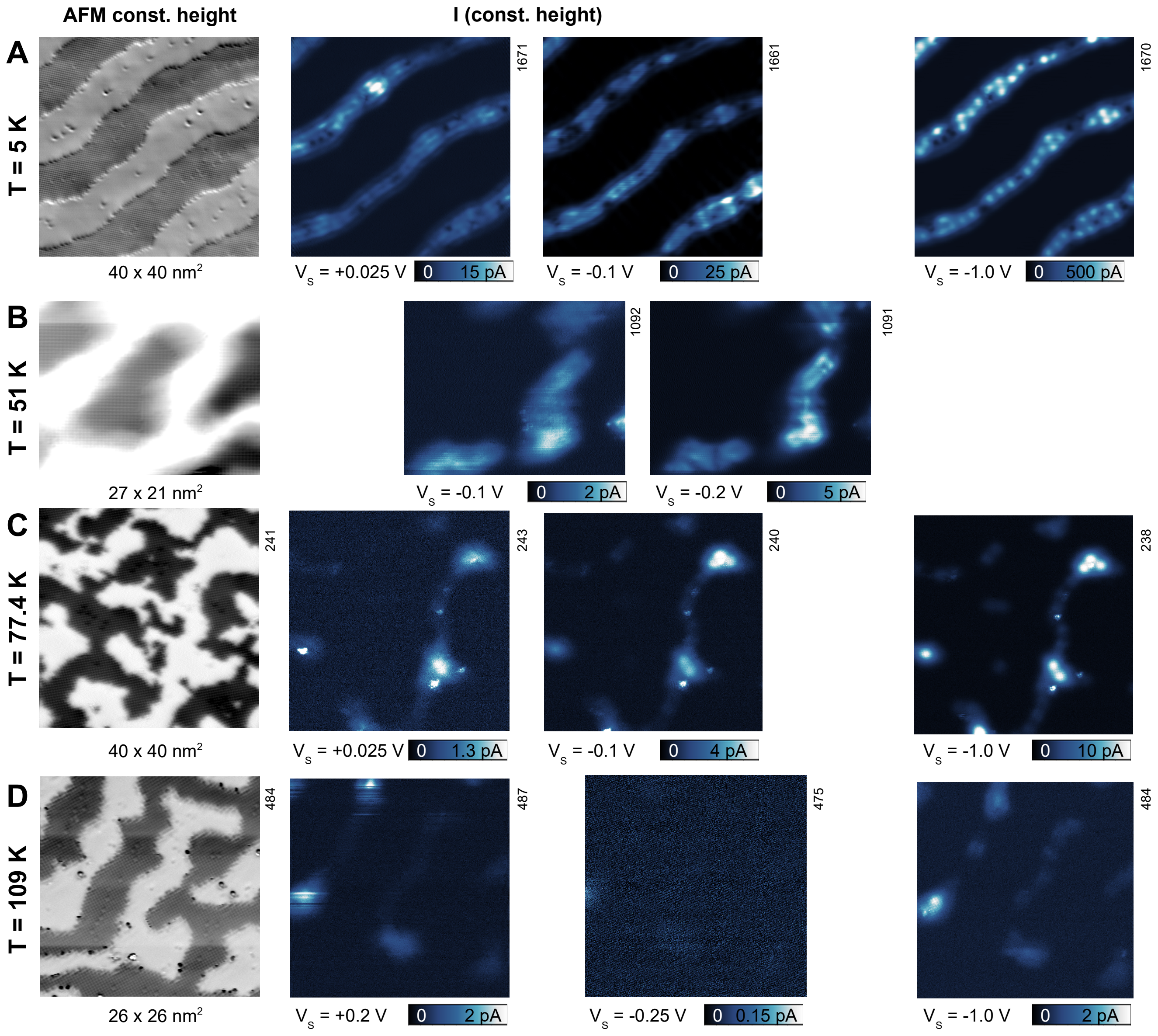

Supplementary Figure 6: **Stability of the CDW** Constant-height images of the tunneling current measured at different temperatures of T =5 K (a), 51 K (b), 77.4 K (c) and 109 K (d). The AFM images in the left column show the layout of terraces in the respective experiments. The tunneling current is recorded for the states directly above and below the Fermi level. Deeper states in the band gap are also included. All data were acquired with a Cu-terminated tip at comparable tip-sample distances. The current images are FFT-filtered to remove the line frequency.

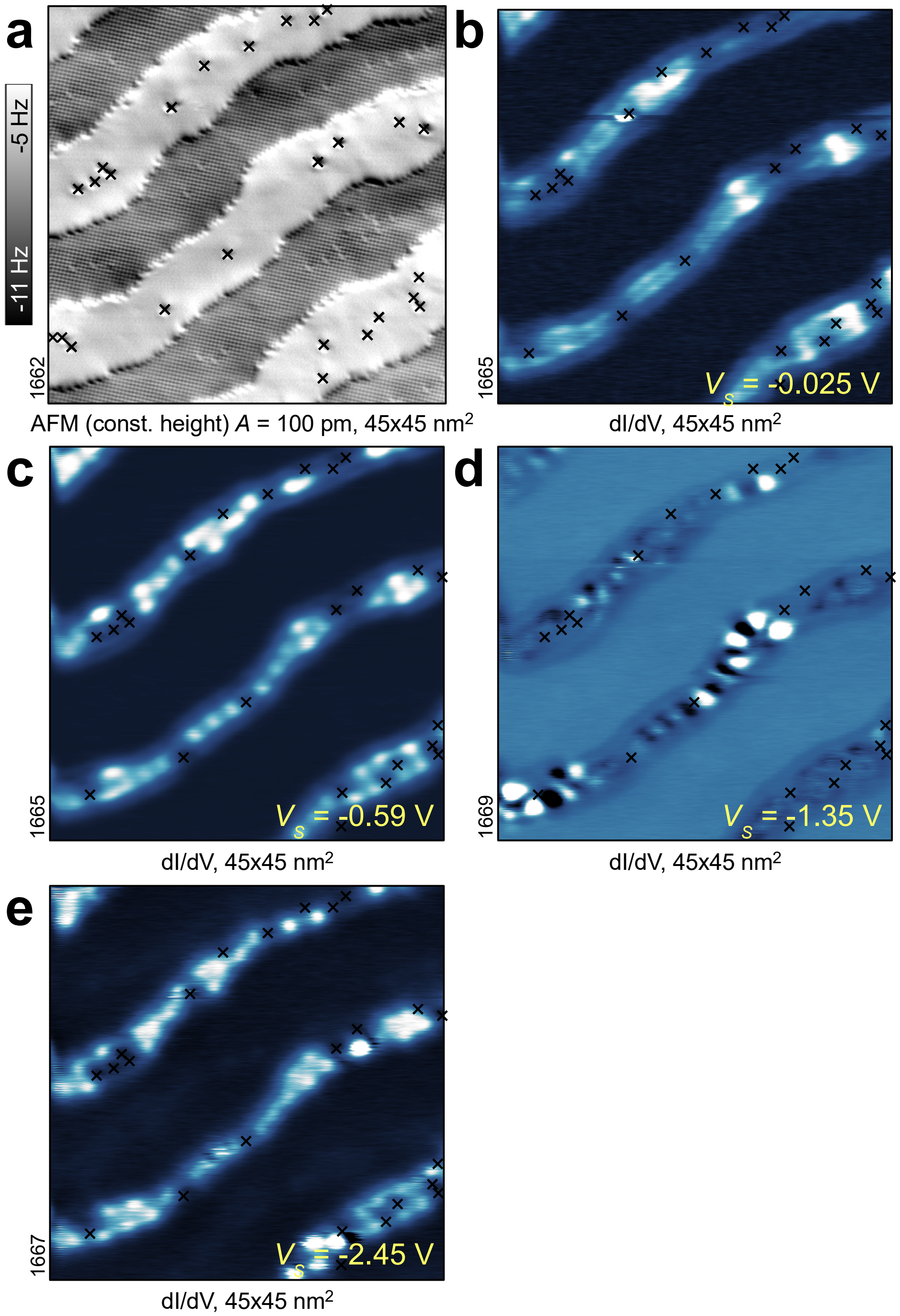

Supplementary Figure 7: **Spatial correlation between polarons and defects**. Data identical to Fig. 1, with positions of structural defects marked by crosses. The maps of LDOS in (b-e) show that the polaron positions are not bound to structural defects in the TaO_2 termination: The correlation is negative, *i.e.*, polarons avoid the defects.

Supplementary Figure 7 shows that the localized polaronic states observed deeper in the band gap are not bound to defects. The data are identical to those shown in Figure 1 of the main text, but the positions of defects on the TaO_2 plane are marked by crosses. The data show that polaronic states are not correlated to defect sites.

Supplementary Note 2. SPIN DEGENERATE 2DEG

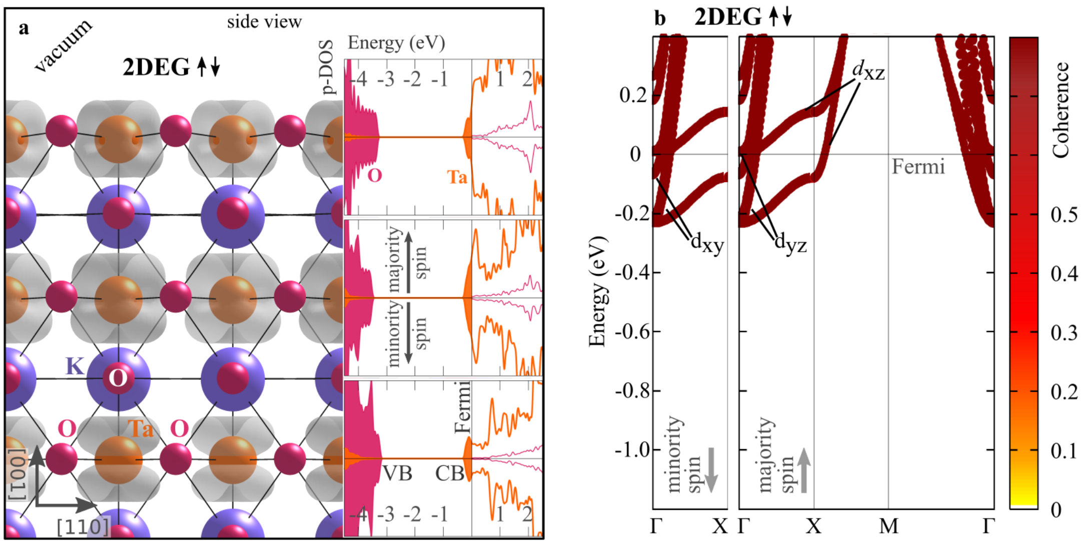

Supplementary Figure 8: **Spin-degenerate 2DEG↑↓ a** Side view of the $KTaO_3$(001) hosting the spin-degenerate 2DEG↑↓ (electronic charge represented by gray clouds), as obtained by the DFT simulations; the insets show the spin-resolved DOS (the labels CB and VB indicate conduction and valence bands, respectively) projected on the corresponding TaO_2 layers, and resolved for the Ta (orange) and O (red) atoms (arbitrary units). **b** Unfolded band structure of the spin-unpolarized 2DEG↑↓ (both spin channels are shown).

Supplementary Figure 8 shows the spin-degenerate 2DEG (labeled as 2DEG↑↓) as obtained in our DFT calculations. Similarly to the spin-polarized 2DEG (here labeled as 2DEG↑↑) discussed in the main text, the spin-degenerate 2DEG↑↓ spreads over surface and shallow sub-surface TaO_2 layers, and it is associated to metallic states at the bottom of the conduction band, and to ferroelectriclike distortions ($\delta_{\mathrm{FE}} = 0.21$ Å, see Supplementary Figure 8a). The layer-resolved density of states shows a symmetric filling of the two spin channels, resulting in a less favorable energy ($\Delta E = +50$ meV per excess electron as compared to the 2DEG↑↓). The spatial distribution of the charge density of the spin-degenerate 2DEG↑↓ (gray cloud in Supplementary Figure 8) appears qualitatively different as compared to the spin-polarized 2DEG↑↑ (shown in Fig. 2 in the main text), due to a different filling of the d orbitals (see Supplementary Figure 8b): In the spin-degenerate 2DEG↑↓ the t_{2g} (d_{xy}, d_{xz} and d_{yz}) band minima are close in energy at the Γ point, and every band crosses the Fermi level [2]. The 2DEG↑↑ shows a similar band structure, but with a full spin polarization (one spin channel is completely empty) favoring occupation of d_{xz} and d_{yz} over d_{xy} (Fig. 4 in the main text).

Supplementary Note 3. DETAILS OF THE FERROELECTRICLIKE DISTORTIONS

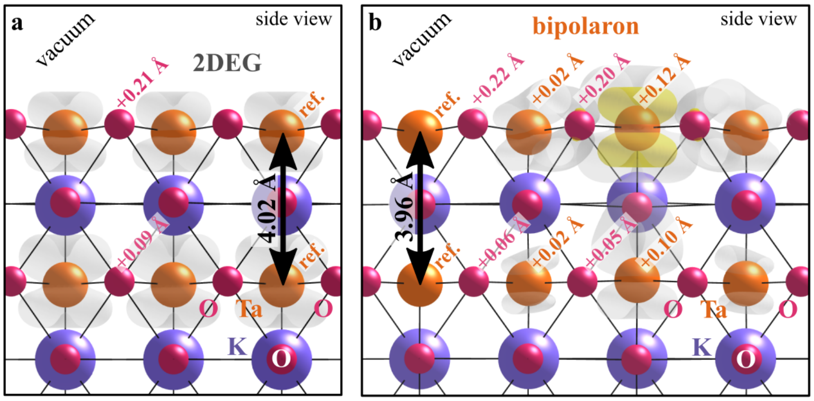

Supplementary Figure 9: **Ferroelectriclike distortions.** Ferroelectriclike distortions of Ta and O atoms in the presence of a 2DEG (panel **a**) and bipolarons (panel **b**). Distortions along [001] are reported with respect to the reference Ta atoms indicated in the Figure.

Supplementary Figure 9 shows the structural details of $KTaO_3(001)$. In the presence of a 2DEG, the TaO_2 layers exhibit evident ferroelectriclike distortions, especially on the surface sites: The O atoms move outwards with respect to the Ta atoms by $\delta_{FE} = 0.21$ Å on the surface layer, and by 0.09 Å on the subsurface layer. The distance between surface and subsurface Ta sites is 4.02 Å.

The formation of bipolarons on the surface leads to electrons strongly localized on few Ta sites. The Ta sites with no polaronic charge show a distance of 3.96 Å from the subsurface TaO_2 layer: Evidently, the lack of conduction-band electronic charge on this site as compared to the 2DEG enhances the attractive electrostatic interaction between the Ta^{5+} atom and the $(KO)^-$ layer.

The bipolaron substantially reduces the local ferroelectriclike distortions, with the bipolaronic Ta site almost aligned with the surrounding O atoms ($\delta_{FE} = 0.08$ Å). Conversely, as discussed in the main text, single-electron polarons localized on surface or subsurface Ta sites affect the surface ferroelectriclike distortions to a smaller extent (distortions of $\delta_{FE} = 0.15$ Å for surface polarons and $\delta_{FE} = 0.23$ Å for subsurface polarons).

Supplementary Note 4. POLARONIC CONFIGURATIONS AND STABILITY

The stability of polarons and bipolarons depends on the polaronic configuration.

Supplementary Figure 10 shows the most stable configuration for a slab with exclusively single-electron polarons, arranged in a $\sqrt{2} \times \sqrt{2}$ pattern on the sub-surface layer. The alternating orbital occupation of d_{xz} and d_{yz} along [110] minimizes the repulsive interaction between polarons ($\Delta E = -230$ meV per excess electron, with respect to the spin-polarized 2DEG).

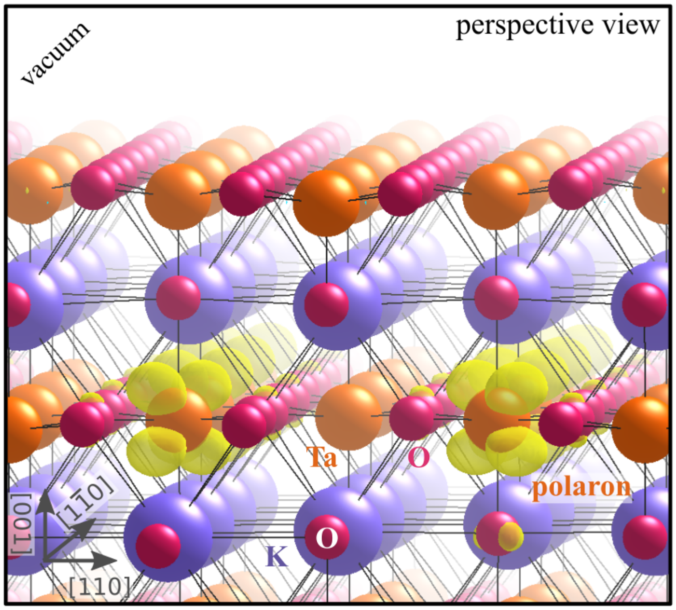

Supplementary Figure 10: **Sub-surface polarons** arranged on a $\sqrt{2} \times \sqrt{2}$ pattern on the sub-surface layer. Yellow color represents the polaronic charge density.

Supplementary Figure 11 shows the two most stable configurations for bipolarons only. Bipolarons aligned along [110] (Supplementary Figure 11a) represent the most stable arrangement (see also Fig. 1c in the main text): They show a high stability ($\Delta E = -180$ meV per excess electron, as described in the main text) and a small overlap of the polaronic charge density on neighboring Ta sites, which occurs between different d orbitals of two bipolarons (d_{xz} and d_{yz}).

Despite being separated by larger distance (8.06 Å instead of 5.70 Å), bipolarons aligned along $\langle 100 \rangle$ directions on the surface (Supplementary Figure 11b) show a lower stability ($\Delta E = -100$ meV): In this case the overlap occurs via the same d orbital (*i.e.*, d_{xz} or d_{yz}) of a neighboring Ta atom, which enhances the inter-polaronic repulsion [4]. The polaronic frustration is indicated also by the shallower and more structured in-gap states (compare the DOS in the insets of Supplementary Figure 11).

The tendency to form bipolarons along [110] suggests the possibility to study the formation of bipolarons by using slabs with smaller lateral dimension along [110]: By adopting $\sqrt{2} \times 4\sqrt{2}$ large slabs, we indeed obtained equivalent results as for the $2\sqrt{2} \times 2\sqrt{2}$ unit cells.

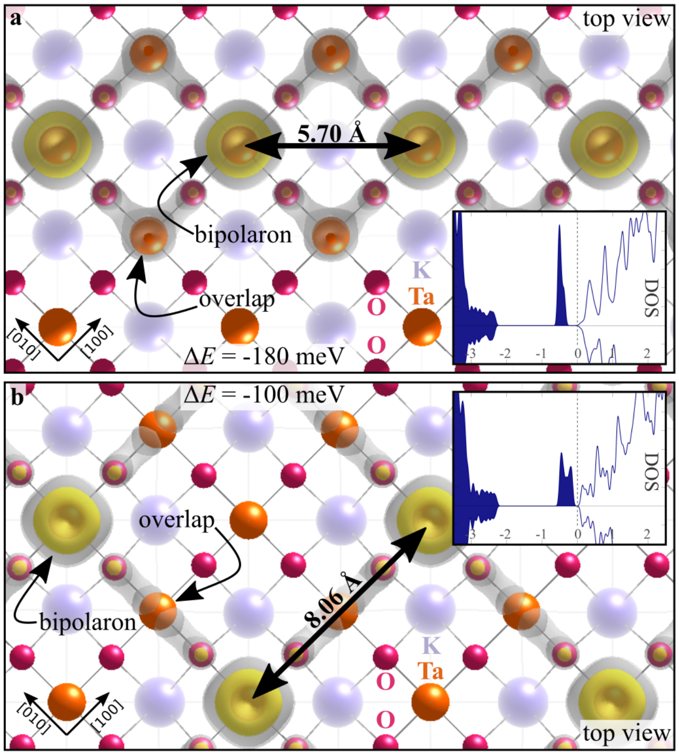

Supplementary Figure 11: **Bipolaron ordering.** Bipolarons arranged along the [110] (panel **a**) or the ⟨100⟩ (panel **b**) directions. Yellow and gray colors represent the polaronic charge density at high and low levels, respectively. Insets show the spin-resolved total DOS for the corresponding bipolaron arrangement.

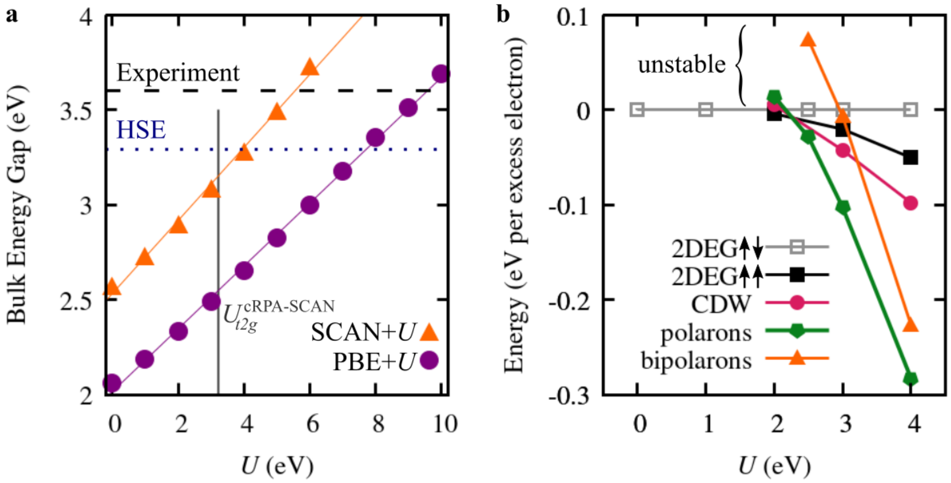

Supplementary Figure 12: **The U parameter.** **a**: Energy band gap of the $KTaO_3$ bulk as obtained using the SCAN (triangles) and PBE (circles) functionals in combination with different values of U. The experimental and HSE06 values are indicated by dashed and dotted horizontal lines, respectively. The $U_{t2g}^{\mathrm{cRPA-SCAN}}$ value (obtained in cRPA calculations for t_{2g} states, *i.e.*, not including the e_g states) is marked by a vertical line. **b**: Stability of the electronic surface states. The energies of bipolarons (triangles), sub-surface polarons (pentagons), CDW (circles) and spin-polarized 2DEG↑↑ (filled squares) are given with respect to the spin-degenerate 2DEG↑↓ (empty squares), as calculated by using various values of U.

Supplementary Note 5. THE U PARAMETER

The DFT calculations were performed using the SCAN [5] functional with an on-site U [6] of 4 eV applied on the d orbitals of Ta atoms (see Methods in the main text). This choice originates from the analysis of the electronic properties shown in Supplementary Figure 12.

Aiming to model in-gap states on the $KTaO_3(001)$ surface, we identified an efficient computational setup able to reproduce the experimental energy gap with reasonable accuracy ($E_{\rm gap}^{\rm exp} \simeq 3.6$ eV [7–10]). Supplementary Figure 12a shows the trend of the energy gap as obtained while varying the value of U in calculations for the bulk $KTaO_3$ primitive cell, using the SCAN and the PBE (from Perdew, Burke, and Ernzerhof [11]) functionals. The SCAN functional performs overall better as compared to PBE: For $U = 4$ eV, SCAN predicts an accurate energy band gap as compared to experiment, and, additionally, reproduces the value of ~ 3.3 eV [12] that we obtained by using hybrid functionals with 25% mixing (HSE, specifically HSE03 [13, 14] and HSE06 [15]), but at considerably different computational cost. The value of $U = 4$ eV agrees well with our constrained random phase calculations (cRPA) that predict a lower limit for the screened Hubbard U of $U_{t2g}^{\rm cRPA-SCAN} = 3.25$ eV (using SCAN wave-functions, a cubic primitive cell, and a $6 \times 6 \times 6$ grid for sampling the reciprocal space). We note that this $U_{t2g}^{\rm cRPA-SCAN}$ value was obtained by calculating cRPA interaction parameters for the t_{2g} states only, since the e_g bands are intermixed with other states; in the DFT+U calculations, the U is instead applied to the full d manifold, thus, canceling the screening of the e_g states. Therefore, a value of U larger than $U_{t2g}^{\rm cRPA-SCAN}$ should be used in the DFT+U calculations: thus, we consider the value reproducing the band gap, $U = 4$ eV, a good choice. It is worth mentioning that the value of U obtained from cRPA calculations provides only an indication for DFT+U calculations, especially when considering surface slabs [16].

At variance with SCAN, results obtained by using PBE are quite contradictory: In fact, the $U_{t2g}^{\rm cRPA-PBE} = 2.95$ eV calculated from PBE wave-functions is unable to reproduce the experimental/HSE energy band gap in the PBE setup. For completeness, we report that the Hubbard J parameter is 0.35 eV for both functionals. We note that we tested even further computational setups, including spin-orbit coupling effects as well as U correction on O atoms up to 8 eV, with no remarkable gain in terms of accuracy with respect to the experiment (not shown in the Figure).

Supplementary Figure 12b shows the energy of different phases of the intrinsic excess charge on the TaO_2 surface, calculated with the SCAN functional and varying the value of U. As evident from the Figure, the emergence of the spin-polarized 2DEG↑↑, the CDW and the (bi)polarons depends on the electronic correlation. As typical for localized states [17], at small values of U, *i.e.*, $U \lesssim 2$ eV, the surface sustains only the homogeneous spin-degenerate 2DEG↑↓. In order to properly model all the states observed in the experiments, larger U values are required: At $U \sim 3$ eV, all the aforementioned states become more stable than the 2DEG↑↓. This is a further evidence of the importance of adopting proper approximations of the electronic correlation [18], but values of U even smaller than $U_{t2g}^{\rm cRPA-SCAN}$ are large enough to obtain stability of CDW and (bi)polaronic states with respect to the homogeneous 2DEG. Approaches beyond DFT+U, such as hybrid functional calculations, are unfortunately computationally unfeasible in the large cells modeling the surface (laterally large slabs to avoid self-interaction of the localized states with their periodic image, and including enough atomic layers to reproduce the ferroelectriclike distortions on the surface). However, we note that it was possible to model the localized states in hybrid functional calculations keeping the ions fixed at the SCAN/PBE+U positions, and using a less accurate setup (energy cutoff of only 400 eV, a coarse $3 \times 3 \times 1$ grid for the sampling of the reciprocal space): In these rough calculations, polarons and bipolarons appear stable, but their electronic properties are affected by the fixed ionic structure [18].

Supplementary Note 6. CDW WAVELENGTH

In the experiment, the CDW coexists with (bi)polarons and shows a periodicity of the charge modulation of 15.5 ± 1.5 Å. The wavelength depends on the degree of band filling. In the DFT simulation of the pure CDW phase (shown in Fig. 3 in the main text), the CDW accommodates all the intrinsic excess charge available on the surface and is characterized by a much shorter wavelength (5.7 Å). Conversely, in the mixed CDW+polaron phase obtained in the 2DEG-to-bipolaron transition (Fig. 5 in the main text), the charge modulation is pinned by the polarons (located in the minima of the wave) and its periodicity is increased to 11.4 Å (the lattice vector of our unit cell), as shown in Supplementary Figure 13. Modeling of CDW with longer wavelength and coexisting with polarons would require a prohibitively larger lateral size of the unit cell.

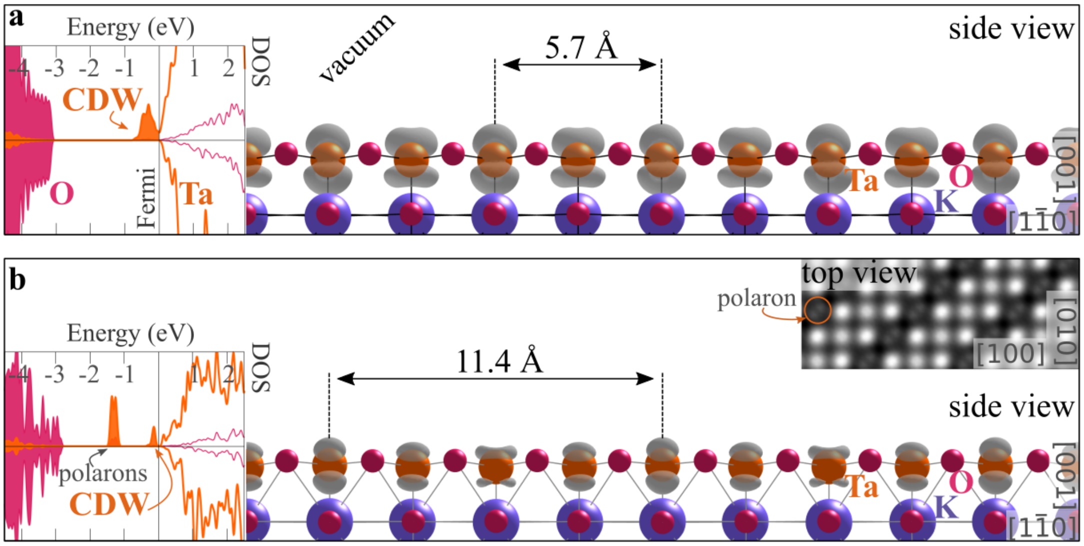

Supplementary Figure 13: **CDW coexisting with polarons.** Side view of the fully CDW phase (**a**) compared to the CDW coexisting with polarons (**b**). The CDW electronic charge is represented by gray clouds, as obtained by integrating the states at the bottom of the conduction band (*i.e.*, the polaronic charge is not included). Insets show the spin-resolved DOS projected on the TaO_2 layers (arbitrary units), and the STM signal as obtained for the CDW states (the position the polaron is indicated by a circle). The wavelengths 5.7 Å and 1.4 Å of the charge modulations are indicated.

Supplementary Note 7. MODELING THE PHASES OF THE EXCESS CHARGE

This section describes how to model in unconstrained DFT+U calculations the electronic surface states arising from the intrinsic excess charge on the TaO_2 termination.

VASP and DFT calculations in general are unable to fully automatically identify the ground state of a given a system. Rather, they convergence to a solution that represents a local minimum in the configuration space, most likely in the proximity of the initial conditions set by the user (*e.g.*, position of ions, local magnetic moments, electron wavefunctions, and more). This allows us to obtain different (structural and electronic) phases by simply choosing suitable initial conditions, with no need for constraints in the calculation. Therefore, we can analyze and compare the properties of all solutions (the local minima), in order to, eventually, indentify the most favorable solution and/or facilitate the interpretation of the experiments.

By adopting this approach, we were able to obtain all the various electronic phases discussed in the main text, *i.e.*, 2DEG↑↓, 2DEG↑↑, CDW and (bi)polarons. Specifically, the 2DEG solutions were obtained by using a slab with an un-distorted lattice (*i.e.*, a lattice with 1×1 periodicity) and randomly initialized wavefunctions with an initial choice for the local magnetic moments of the atoms in the system: 0 μ_B for all atoms to obtain the 2DEG↑↓, and ~ 1 μ_B for the Ta atoms to obtain the 2DEG↑↑. We obtained both 2DEG solutions in the $2\sqrt{2} \times 2\sqrt{2}$ slab even when symmetry constraints (typically used to speed up the calculations) were removed.

The (bi)polarons were modeled with one of the standard approaches for localizing polaronic states [17, 19]: (*i*) we initially introduced (manually) local lattice distortions (elongated Ta-O bonds) around the Ta ion selected as hosting site for the (bi)polaron; (*ii*) we set the initial local magnetic moments to 0 μ_B for all atoms except the Ta atoms meant to host a polaron (~ 1 μ_B) or a bipolaron (~ 2 μ_B). (*iii*) finally, we run a calculation to relax the lattice and electronic structures. It might be useful to mention that there are other possible strategies for the formation of polarons in materials modeling (*e.g.*, intermediate calculations with large U values on the localization sites, or with local atomic substitution, [17], or by constraining the density matrix of specific sites to the polaronic occupation [20], or via molecular dynamics calculations [21], and more).

The CDW was modeled by using the undistorted lattice (the 1×1 lattice of the 2DEG) and by initializing the wafunctions to those obtained for the sub-surface polarons in the $\sqrt{2} \times \sqrt{2}$ arrangement. While polaronic and CDW wavefunctions are different from each other (see the energy bands in Fig. 4 in the main text that shows also different orbital character occupations), the CDW charge shares the same periodicity as this particular arrangement of polarons. The combination of the 1×1 lattice with the $\sqrt{2} \times \sqrt{2}$ charge modulation makes the calculation converge to the CDW solution (the polaronic solution becomes unstable in the very first steps of the calculations in the absence of *ad-hoc* structural distortions).

Finally, it is worth noting that the investigation of the phase transitions shown in Fig. 5 of the main text, where we progressively moved the ions from one structure to another, led to spontaneous changes of the electronic phases: for example, the CDW was obtained as a spontaneous result in the mixed CDW+polaron phase (shown in Supplementary Figure 13) while the lattice was progressively distorted between the 2DEG structure and the bipolaronic phase.

---

[1] P. D. King, R. H. He, T. Eknapakul, P. Buaphet, S. K. Mo, Y. Kaneko, S. Harashima, Y. Hikita, M. S. Bahramy, C. Bell, Z. Hussain, Y. Tokura, Z. X. Shen, H. Y. Hwang, F. Baumberger, and W. Meevasana, Physical Review Letters **108**, 117602 (2012).

[2] A. F. Santander-Syro, C. Bareille, F. Fortuna, O. Copie, M. Gabay, F. Bertran, A. Taleb-Ibrahimi, P. Le Fèvre, G. Herranz, N. Reyren, M. Bibes, A. Barthélémy, P. Lecoeur, J. Guevara, and M. J. Rozenberg, Physical Review B - Condensed Matter and Materials Physics **86**, 121107 (2012).

[3] M. Spera, A. Scarfato, Á. Pásztor, E. Giannini, D. R. Bowler, and C. Renner, Physical Review Letters **125**, 267603 (2020).

[4] M. Reticcioli, M. Setvin, M. Schmid, U. Diebold, and C. Franchini, Physical Review B **98**, 045306 (2018).

[5] J. Sun, R. C. Remsing, Y. Zhang, Z. Sun, A. Ruzsinszky, H. Peng, Z. Yang, A. Paul, U. Waghmare, X. Wu, M. L. Klein, and J. P. Perdew, Nature Chemistry **8**, 831 (2016).

[6] S. Dudarev and G. Botton, Physical Review B - Condensed Matter and Materials Physics **57**, 1505 (1998).

[7] G. O. Deputy and R. W. Vest, Journal of the American Ceramic Society **61**, 321 (1978).

[8] G. E. Jellison, I. Paulauskas, L. A. Boatner, and D. J. Singh, Physical Review B **74**, 155130 (2006).

[9] S. H. Wemple, Physical Review **137**, A1575 (1965).

[10] A. Frova and P. J. Boddy, Physical Review **153**, 606 (1967).

[11] J. P. Perdew, K. Burke, and M. Ernzerhof, Physical Review Letters **77**, 3865 (1996).

[12] B. Modak and S. K. Ghosh, The Journal of Physical Chemistry C **120**, 6920 (2016).

[13] J. Heyd, G. E. Scuseria, and M. Ernzerhof, The Journal of Chemical Physics **118**, 8207 (2003).

[14] J. Heyd and G. E. Scuseria, The Journal of Chemical Physics **121**, 1187 (2004).

[15] A. V. Krukau, O. A. Vydrov, A. F. Izmaylov, and G. E. Scuseria, The Journal of Chemical Physics **125**, 224106 (2006).
[16] E. Şaşıoğlu, C. Friedrich, and S. Blügel, Physical Review Letters **109**, 146401 (2012).
[17] M. Reticcioli, U. Diebold, G. Kresse, and C. Franchini, in Handbook of Materials Modeling (Springer International Publishing, Cham, 2020) pp. 1–39.
[18] M. Reticcioli, U. Diebold, and C. Franchini, Journal of Physics: Condensed Matter **34**, 204006 (2022).
[19] T. D. Pham and N. A. Deskins, Journal of Chemical Theory and Computation **16**, 5264 (2020).
[20] J. P. Allen and G. W. Watson, Physical Chemistry Chemical Physics **16**, 21016 (2014).
[21] A. L. Shluger and A. M. Stoneham, Journal of Physics: Condensed Matter **5**, 3049 (1993).
